# Slow water dynamics in dehydrated human Jurkat T cells evaluated by dielectric spectroscopy with the Bruggeman–Hanai equation†

**DOI:** 10.1039/d3ra02892e

**Published:** 2023-07-11

**Authors:** Hiroaki Matsuura, Kiyoshi Takano, Ryo Shirakashi

**Affiliations:** a Institute of Industrial Science, The University of Tokyo Meguro Tokyo 153-8505 Japan matsuura@iis.u-tokyo.ac.jp; b Research Fellow of the Japan Society for the Promotion of Science Chiyoda Tokyo 102-0083 Japan

## Abstract

The picosecond orientational dynamics of intracellular water was measured by dielectric spectroscopy, with the aim of revealing the effects of cryoprotective agents (CPAs) on biological cells. As a first step, Jurkat cells (human T lymphocyte cells) suspended in aqueous sucrose solutions of different concentrations ranging from 0.3 M (isotonic) to 0.9 M (hypertonic) were examined at 25 °C with a frequency range up to 43.5 GHz. The Bruggeman–Hanai equation was employed to obtain a cellular dielectric spectrum without extracellular contributions from the measured complex permittivity of the cell suspensions. By analyzing the γ process around 10^10^ Hz based on the Debye relaxation function, two types of water (bulk-like water and hydration water with slower molecular dynamics) were observed. An increase in the fraction of intracellular slower water was observed in the dehydrated cells which had a highly concentrated environment of biomolecules.

## Abstract 2

The picosecond orientational dynamics of intracellular water was measured by dielectric spectroscopy, with the aim of revealing the effects of cryoprotective agents (CPAs) on biological cells.

## Graphical abstract

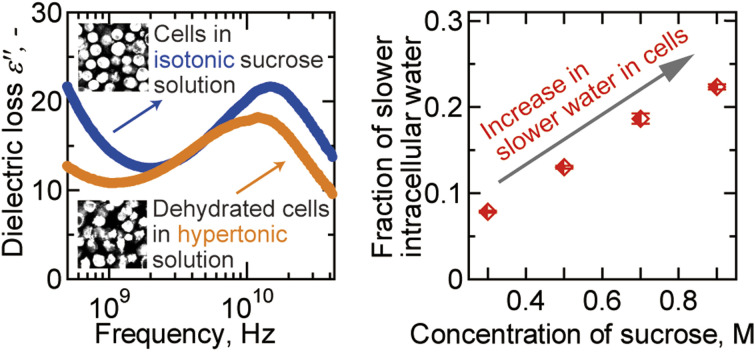

## 1 Introduction

Water in cells is not just a passive matrix of “life's solvent”, but an active participant in various chemical and biophysical processes, including electron and proton transfer, protein folding, and enzyme catalysis.^1,2^ Since up to 400 mg ml^−1^ of macromolecules are contained in cytoplasm,^1^ the structural and dynamic characteristics of water in this extremely crowded environment would be quite different from those of pure water. Evaluating the properties of intracellular water molecules is important not only for the fundamental understanding of biosystems but also for applications such as the cryopreservation of biological cells. In cryopreservation, an understanding of the dynamics of water molecules in cells is crucial for avoiding the fatal freezing injuries related to intracellular ice formation (IIF).^3,4^ Because the probability (or kinetics) of ice formation is strongly dependent on the amount of intracellular water, the appropriate cellular dehydration is important to reduce IIF.^5^ Cryoprotective agents (CPAs), which affect the plasma membrane transport of water and ice nucleation, are added before cooling to reduce cellular damage,^6^ but this operation is generally based on empirical knowledge and there is clear room for optimization. For the efficient design of new CPAs and the development of optimized cryopreservation protocols, an understanding of the biophysical properties of cells is important. A number of experimental techniques have been employed to reveal intracellular water molecular dynamics, including optical Kerr decay microspectroscopy,^7^ quasi-elastic neutron scattering (QENS),^8–12^ spin relaxation nuclear magnetic resonance (NMR),^12–15^ ultrafast vibrational spectroscopy and dielectric spectroscopy,^16^ but consensus has not been reached about the intracellular water dynamics.

In the present study, we investigated the molecular dynamics of water in dehydrated mammalian cells by dielectric spectroscopy (DS). This technique can evaluate the collective orientational motion of the dipole moments of molecules by measuring the polarization induced by the electric-field.^17,18^ Although DS has been widely employed in the investigation of water dynamics in aqueous solutions of biomolecules,^17^ measurements of the picosecond dynamics of intracellular water have been very limited. Tros *et al.*^16^ measured the orientational dynamics of water in *E. coli*, *Bacillus subtilis*, and *S. cerevisiae* yeast by DS. However, there have been no DS studies focusing on the effect of CPAs on water dynamics in mammalian cells. As a first step to understanding the properties of intracellular water in the cryopreservation process, we performed dielectric spectroscopy on Jurkat cells (human T lymphocyte cells) suspended in sucrose aqueous solutions of different concentrations (isotonic to hypertonic) at a temperature of 25 °C with the frequency range up to 43.5 GHz. Since sucrose is one of the non-membrane-permeable CPAs that are scarcely delivered into cells,^6^ cells are dehydrated and shrink in its hypertonic solutions. The dielectric spectrum of cell suspensions above GHz range includes the contribution of both intra- and extracellular water. Feldman *et al.*^19–21^ isolated the dielectric spectrum of cytoplasm based on the Kraszewski's equation,^22^ with the assumption that cell suspensions behave as a sum of infinite number of thin water and dry substance layers. It should be noted that Kraszewski's model^22^ was developed for the analysis of clay–sand slurry which has dielectric properties different from cell suspensions.^23^ In the present study, the Bruggeman–Hanai (BH) equation,^24^ which describes the permittivity of homogeneously distributed spherical particles, has been employed. By decomposing dielectric spectra based on the multi-Debye relaxation model,^17^ we evaluated the effect of cell dehydration on the molecular dynamics of intracellular water.

## 2 Experimental section

### 2.1 Sample preparation

Sucrose was supplied by Wako Co. (Japan) and used without further purification. Aqueous solutions of sucrose were prepared with ultrapure water from water purification system (Simplicity UV, Merck, Germany). The concentrations of sucrose (*c*_suc_) were 0.3, 0.5, 0.7 and 0.9 M, which correspond to osmolarities of 308 (isotonic), 518, 732, and 949 (hypertonic) mOsm, respectively.^25^ Jurkat cells were obtained from the Riken BioResource Research Center (Tsukuba, Japan) and cultured at 37 °C with 5% CO_2_ in RPMI-1640 culture medium (Gibco, USA) supplemented with 10% fetal bovine serum (FBS; Gibco). After cells in logarithmic growth phase were harvested by centrifugation and rinsed with phosphate-buffered saline (PBS), cells were suspended in aqueous sucrose solution and centrifuged again. A concentrated cell suspension for measurement was obtained by removing the supernatant. A part of the supernatant was kept for the measurement of dispersion medium, which is required for analysis based on the BH equation.

### 2.2 Dielectric spectroscopy

The dielectric spectra were obtained using a vector network analyzer (N5224A, 10 MHz to 43.5 GHz: Agilent Technologies, USA) and two impedance analyzers (4294A, 40 Hz to 110 MHz: Agilent Technologies; and E4991B, 1 MHz to 3 GHz: Keysight Technologies).^26,27^ These three instruments were connected to an open-ended coaxial probe with a diameter of 1.2 mm (Coax, Japan) *via* a multiport coaxial switch (8767M, DC to 50 GHz: Keysight Technologies) and coaxial flexible cable (FC-182, up to 40 GHz: Flexco Microwave, USA), as shown in Fig. S1 in the ESI.† The temperature of the sample was maintained at 25.0 ± 0.1 °C by circulating water in the holder of the test fixture from a thermostatic bath (TRL-108H; Thomas Kagaku, Japan). In the experiments, the dielectric spectra of dispersion medium (supernatant of cell suspension) and cell suspensions were obtained after the measurement of calibration standards with known dielectric properties, including water, 2-propenol, ethylene glycol, and air.^26–28^

The imaginary part *ε*′′ (dielectric loss) of the complex permittivity, ε* = *ε*′ − *iε*′′ (*i* is the imaginary unit), is calculated from the experimentally obtained real part permittivity *ε*′ using the Kramers–Kronig (KK) relation.^27^ This KK transform from the real part *ε*′ allows us to eliminate the contribution of static electrical conductivity (ohmic loss), which sometimes severely overlaps the dielectric relaxation of water.

### 2.3 Analysis of the dielectric spectrum of cells with the Bruggeman–Hanai (BH) equation

To obtain the dielectric properties of the cells themselves, we used the Bruggeman–Hanai (BH) equation developed by Hanai^24^ for concentrated suspensions of spherical particles based on Bruggeman's effective medium approximation.^29^ By the BH equation, the complex permittivity of dispersion medium 
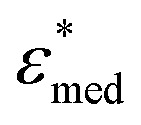
, cell suspension 
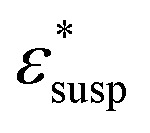
, and cells (dispersed particles) 
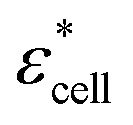
 can be expressed as eqn (1).^24^1
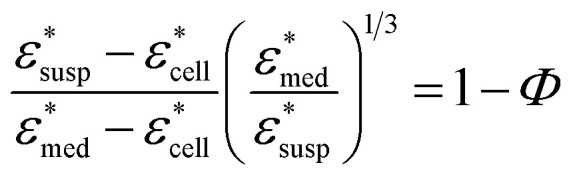
Here, *Φ* is the volume fraction of cells in the sensing volume. The BH equation has been successfully applied to the analysis of various colloidal dispersions, including suspensions of liposome, erythrocytes, and yeast cells, over a wide range of volume fractions up to 0.8.^30^ However, in most studies the dielectric relaxation due to the interfacial polarization at the membrane (the Maxwell–Wagner effect)^17^ in the MHz frequency range was examined by the BH equation, where intracellular permittivity and conductivity are assumed to be constant. Recently, Takei's group^31^ employed the BH equation to extract the complex permittivity of yeast cells up to 3 GHz, where the dielectric relaxation originating from water around 10^10^ Hz has not been examined. In the present study, the dielectric spectrum of cells up to 43.5 GHz was obtained by the BH equation, which enabled analysis of the water dielectric relaxation. The volume fraction *Φ* in eqn (1) was measured independently from the DS experiments, by optical observation of cell suspensions with a confocal laser scanning fluorescence microscope (Fluoview FV-300; Olympus, Japan). After measuring 
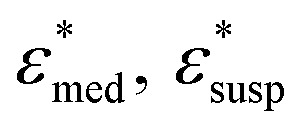
, and *Φ*, the complex permittivity of cells 
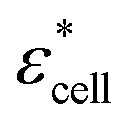
 can be derived by eqn (1).

The dielectric relaxations were analysed by decomposing dielectric loss spectra into the sum of the Debye relaxations (eqn (2))^17,26^ by the least square approximation.2
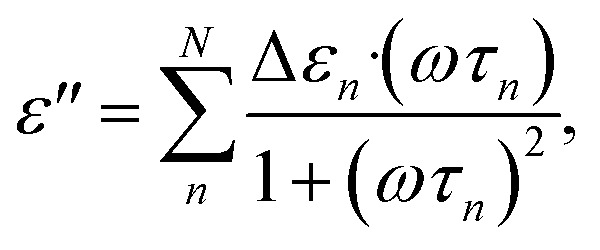
where *ω* is the angular frequency, Δ*ε*_*n*_ is the relaxation strength, and *τ*_*n*_ is the relaxation time for the *n*-th term. For the dielectric relaxation due to the orientational polarizations, Δ*ε*_*n*_ and *τ*_*n*_ characterize the molar concentration and the molecular dynamics, respectively. In this study, the number of relaxation terms *N* in the right hand of eqn (2) and (3) was determined based on the Bayesian information criterion (BIC), in which *N* for the lowest value of BIC is preferred in the model selection.^32^ It is known that the broadening of the dielectric relaxation is observed for aqueous solutions and the use of empirical Cole–Cole type function (eqn (3)) is prevalent in the analysis of biological systems,^17^3
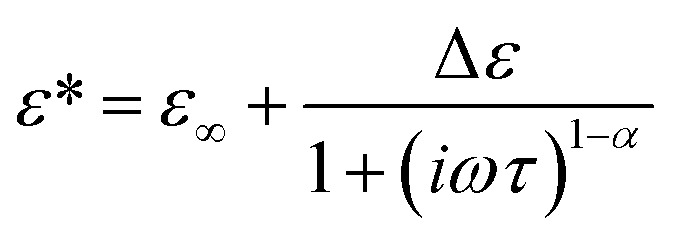
in which *α* is the broadening factor and *ε*_∞_ is the permittivity at the high frequency limit. This function reduces to the Debye expression when *α* = 0. Although progress has been made recently on the physical interpretation of *α*, it remains to be established.^17^ On the other hand, the Debye-type relaxation function can be derived by the Langevin equation and *τ* in that function can be clearly connected to the rotational motion of molecular dipole moments. Thus, we adopt the Debye-type function for the analysis of the dielectric spectra in the present study.

## 3 Results and discussion

**Fig. 1:**
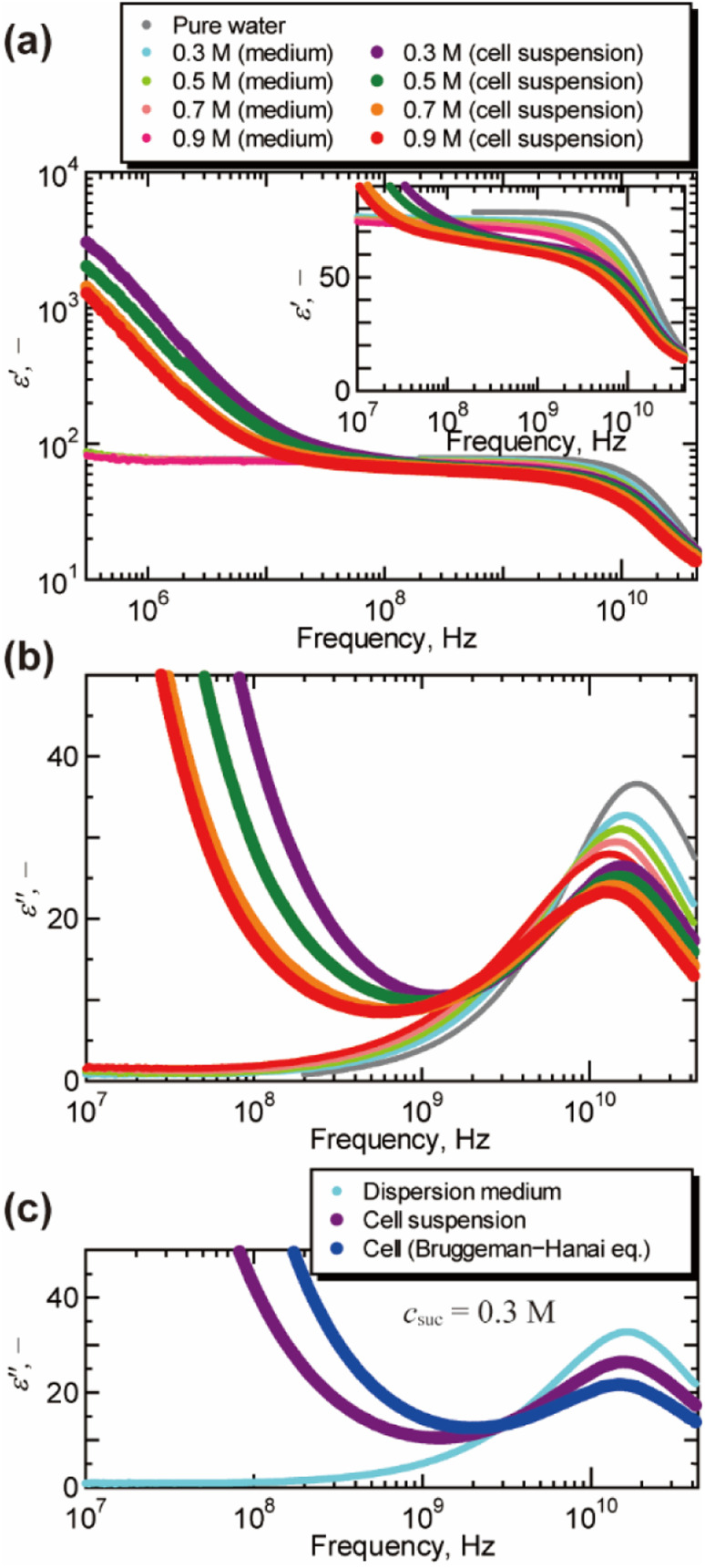
Dielectric spectra of cell suspensions, dispersion medium, and cells at 25 °C. (a) The real part of the relative permittivity *ε*′ after subtracting the contribution of the electrode polarization (*ε*_EP_ = *Aω*^−*λ*^, in which *ω* is the angular frequency, *A* and *λ* are the constants of the system^17,26^). The inlet shows a magnified view for the frequency above 10^7^ Hz. (b) The imaginary part of the relative permittivity *ε*′′ obtained by the Kramers–Kronig transform of the real part *ε*′. For the raw data of dielectric loss by the vector network analyser, see Fig. S2 in the ESI.† (c) The dielectric loss spectrum of cells suspended in aqueous sucrose solution of 0.3 M obtained by the Bruggeman–Hanai equation.

Fig. 1 shows the dielectric spectra of cell suspensions and dispersion medium. A dielectric relaxation observed around 10^10^ Hz is known as the γ process, and is attributed to the collective orientational motion of the dipole moments of water molecules.^17^ A large relaxation found in the spectra of cell suspensions in the frequency range of 10^4^ to 10^8^ Hz is called the β process, and is caused by the Maxwell–Wagner effect.^17^ As shown in Fig. 1(b), the peak of the γ process in the dielectric loss spectra moved to the lower frequency as the concentration of sucrose increased. This peak shift indicates the increase in the dielectric relaxation time, which leads to the retardation of water molecular dynamics under the oscillating electric field. The γ process was also found in the dielectric loss spectra of cells obtained by the BH equation, as shown in Fig. 1(c) for *c*_suc_ = 0.3 M (for other sucrose concentrations, see Fig. S3 in the ESI†). The volume fractions observed by confocal microscopy (Fig. S4 in the ESI†) were 0.54 ± 0.03, 0.49 ± 0.01, 0.45 ± 0.03, and 0.45 ± 0.01 for *c*_suc_ = 0.3 M, 0.5 M, 0.7 M, and 0.9 M, respectively. It should be noted that the BH equation was derived by Hanai based on the Bruggeman's approximation without solving the particle-to-particle interactions rigorously.^17^ Numerical studies^17,33,34^ demonstrated that the interactions between cells have significant effects on the β process in the frequency range of 10^4^ to 10^8^ Hz. However, it was not necessary to consider the effects of the interactions between cells in the present study, since this work was focused on the γ process in the higher frequency above 10^8^ Hz. In addition, the Maxwell–Wagner effect due to the membrane of organelles^17^ can also be ignored in frequency range of γ process.

**Fig. 2:**
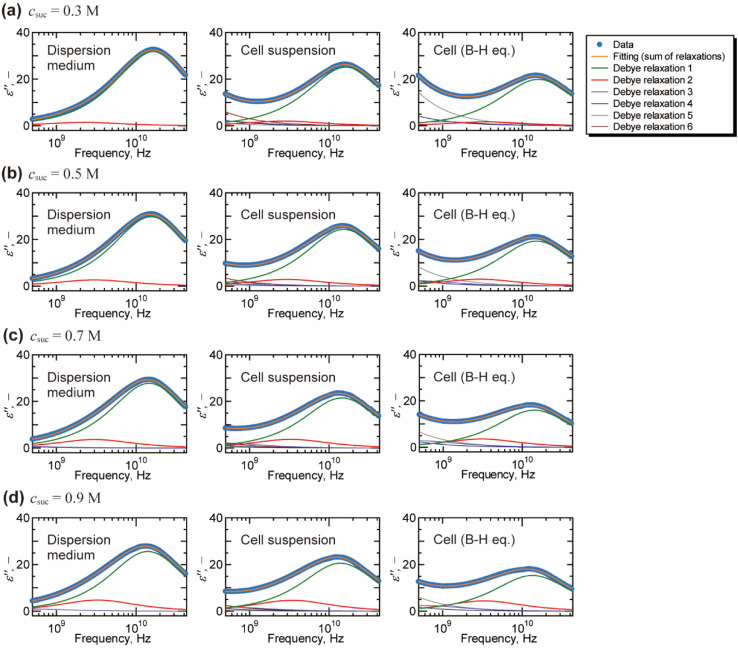
Decomposition of the dielectric loss spectra based on the Debye relaxation functions (eqn (2)) for dispersion medium, cell suspension, and cells (by Bruggeman–Hanai equation). The fitting analysis was performed on the dielectric loss spectra with the frequency range of 10 MHz to 43.5 GHz. The fitting results above 500 MHz are shown for the visual convenience of γ process. Only relaxations 1 and 2 for γ process are examined in this study which focuses on the molecular dynamics of water. Although relaxations 3, 4, 5, and 6 contain information of low frequency phenomena, such as Maxwell–Wagner effect, we do not discuss them in the present study. Initial values and the repeatability of the fitting results are shown for the spectrum of cell suspension (*c*_suc_ = 0.9 M) in Table S2 in the ESI.†

Fig. 2 shows a typical result of decomposing spectra into the Debye relaxations by eqn (2) for the dispersion medium, cell suspensions, and cells by BH equations. The γ process was well described by the sum of two Debye relaxations, *i.e.*, the faster (green) and the slower (red) ones. Note that γ process for cells cannot be expressed well by single Debye relaxation, as shown in Fig. S5 in the ESI.† Example of BIC for each number of relaxation *N* and values of *N* determined based on BIC are shown in Fig. S6 and Table S1,† respectively. Similar decompositions of the γ process into two Debye relaxations were obtained in DS studies of mono and disaccharide aqueous solutions.^35–37^ Faster and slower processes, which we call the relaxations 1 and 2, are attributed to the collective reorientation of bulk-like water molecules and water molecule with retarded reorientational dynamics (hydration water), respectively. The two types of intracellular water have also been observed in QENS and NMR studies.^9–11,13^ Note that two relaxation processes were found by the dielectric spectroscopy for cell suspensions by Tros *et al.*,^16^ in which the Cole–Cole type equations were used for the fitting to the dielectric spectrum with a frequency range of 760 MHz to 70 GHz. Nevertheless, it is difficult to eliminate the ohmic loss dominating the measured dielectric loss spectrum below a few GHz frequency that overlaps with the dielectric relaxation originated in other relaxation modes in lower frequency range.^22^ In the present study, we circumvented this problem of the ohmic loss contributions by analysing the dielectric loss spectrum obtained by the real part of the complex permittivity based on the Kramers–Kronig relations,^27^ which enabled evaluation of the retarded water dynamics. Although even slower relaxations known as the δ process were observed in the frequency range below 1 GHz, we do not discuss them in the present study, since there is no consensus about the origin of the δ process.^17,38^ A computational study by Braun *et al.*^38^ showed that cross-correlation between water and protein dipoles plays a key role in the δ process for the dielectric spectrum of aqueous protein solutions. This type of cross contribution might be included in the δ process for the spectrum of cells where biological macromolecular dynamics occupy a considerable part.

**Fig. 3:**
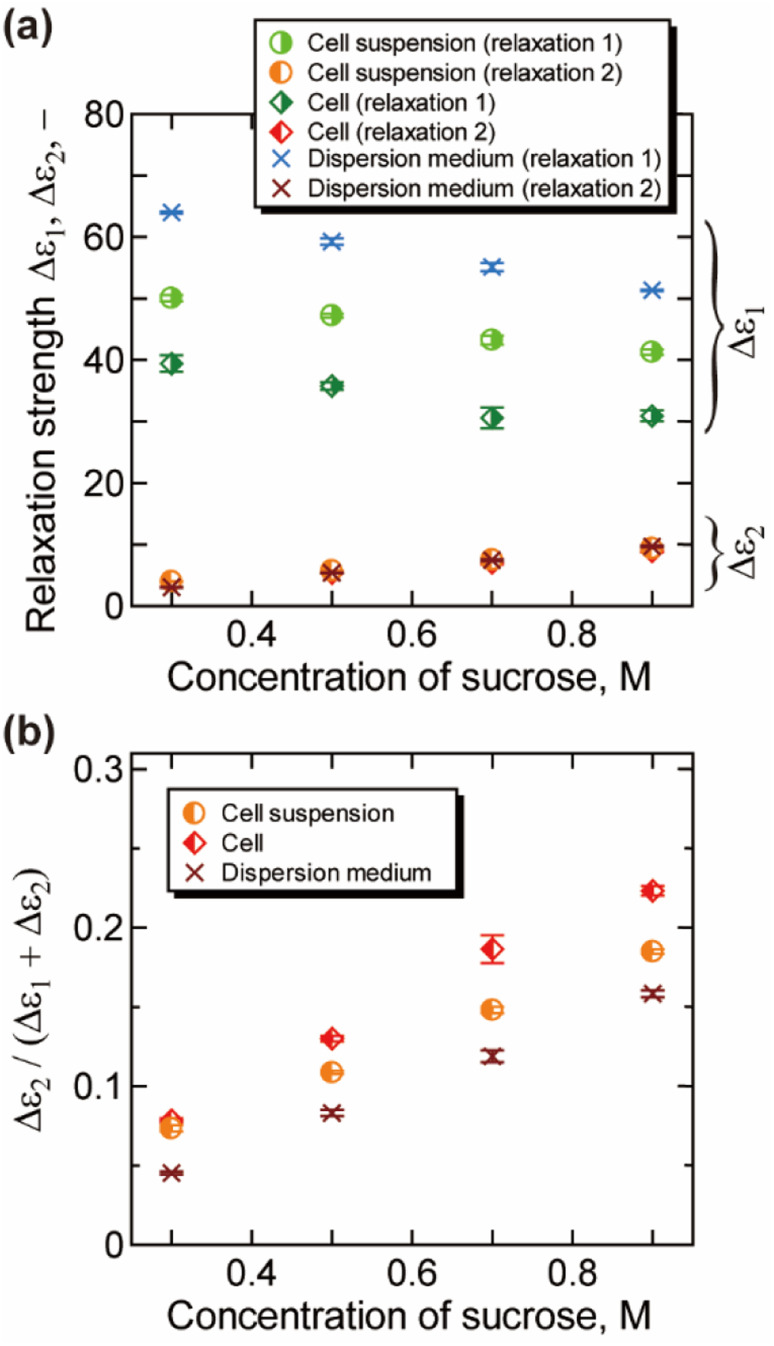
Relaxation strength obtained by the analysis of the γ process (25 °C). (a) The relaxation strength for the bulk-like water Δ*ε*_1_ and slower water Δ*ε*_2_. (b) The ratio of slower water Δ*ε*_2_/(Δ*ε*_1_ + Δ*ε*_2_). Error bars are the standard deviation. Contribution of the uncertainty of the measurement of volume fraction is combined for the results of cells.

**Fig. 4:**
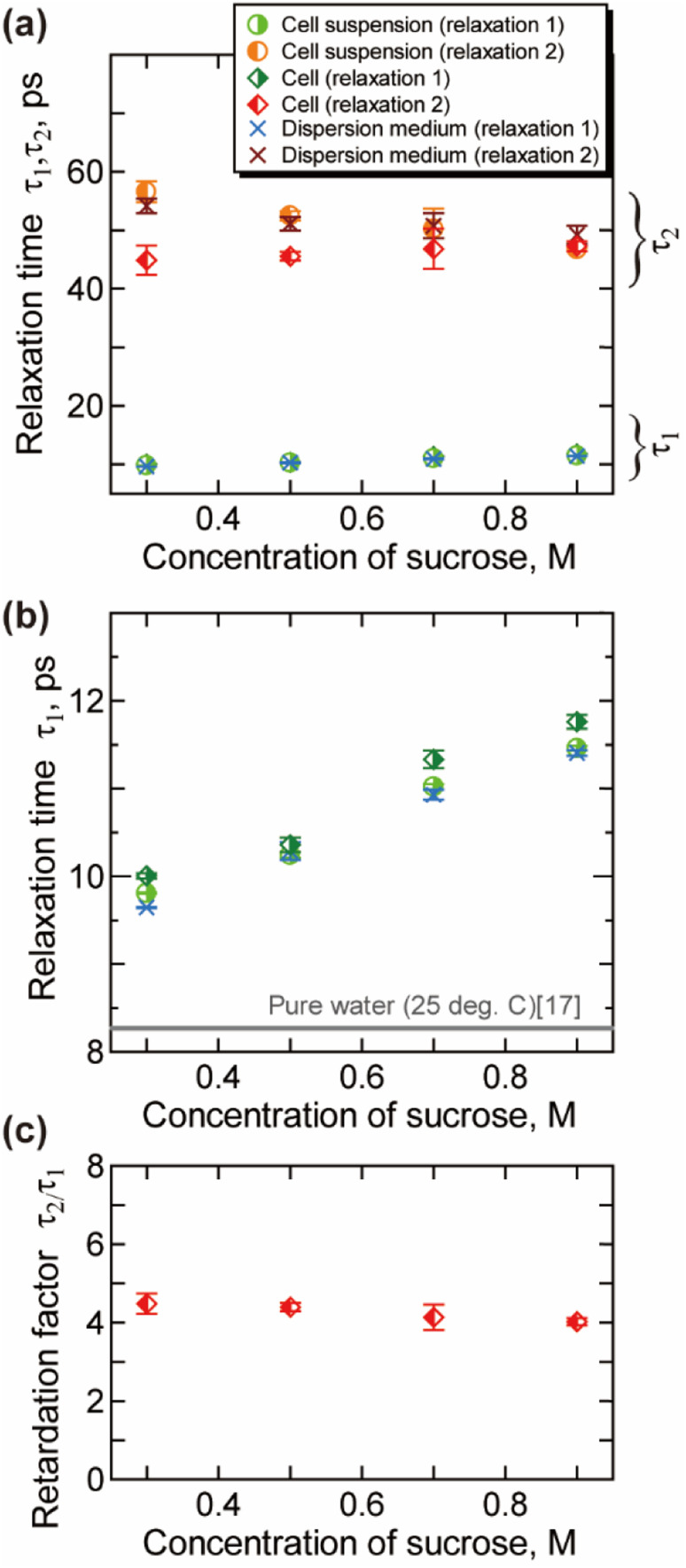
Relaxation time obtained by the analysis of the γ process (25 °C). (a) The relaxation time for the bulk-like water *τ*_1_ and slower water *τ*_2_. (b) The magnified view of (a) for *τ*_1_. (c) The retardation factor *τ*_2_/*τ*_1_ for the intracellular water. Error bars are the standard deviation.

The relaxation strength and the relaxation time obtained by the decomposition of the γ process (relaxations 1 and 2) are plotted in Fig. 3 and 4, respectively. For dispersion medium, the relaxation strength of hydration water Δ*ε*_2_ increased with the increase of the concentration of sucrose, while that of bulk water Δ*ε*_1_ decreased. Similar trends were observed for Δ*ε*_1_ and Δ*ε*_2_ of cell suspensions and cells. Note that the results for cell suspensions contain contributions of both intra- and extracellular water, while the contribution of extracellular water was excluded for the results of cells by the BH equation. The ratio of hydration water Δ*ε*_2_/(Δ*ε*_1_ + Δ*ε*_2_) for cells in Fig. 3(b) is higher than that for dispersion medium and cell suspensions. This suggests that the hydration water makes up a larger part of the water in cells, reflecting the retardation of the water dynamics under more “crowded” cell conditions. The ratio Δ*ε*_2_/(Δ*ε*_1_ + Δ*ε*_2_) was changed by the cellular dehydration to a value in the range of 8% to 22%. These values are comparable to the fraction of intracellular slow water without dehydration by NMR and QENS studies on erythrocytes (10%),^10^*Escherichia coli* (15%),^13^*Haloarcula marismortui* (15%),^13^ yeast (21%),^11^ and Glioma-9L (7%).^11^

The relaxation time for the faster process *τ*_1_ shown in Fig. 4(b) becomes longer with the increase of the *c*_suc_. The relaxation time *τ*_1_ in cells is slightly longer than that in the extracellular dispersion medium, especially at a higher hypertonicity. As demonstrated in Fig. 4(c), no significant dehydration-induced change was observed in the retardation factor *τ*_2_/*τ*_1_ of cells, which is comparable to the rotational retardation factor of 5 for *E. coli* and yeast cells by the QENS study of Piazza *et al.*^11^ Considering the result for the relaxation strength, it was revealed that the hydration water with slow dynamics accounts for a larger part of water in the dehydrated cells under crowded conditions without a prominent change in the retardation factor for the relaxation time *τ*_2_/*τ*_1_. Note that an increase in the broadening factor *α* was observed for dehydrated cells by the curve fitting to the γ process based on a single Cole–Cole relaxation (eqn (3)) as shown in Fig. S8(c) in the ESI,† although there is relatively large deviation between the data and fitting curves (Fig. S7†). This broadening has been expressed by the sum of two Debye-type relaxations in the present study.

Cytoplasm in a dehydrated cell is more concentrated than that in a cell in isotonic condition. Robles-Hernández *et al.*^39^ measured concentrated aqueous solutions of single-chain polymer nanoparticles, which mimic cellular environments, by the dielectric spectroscopy. The Spanish group^39^ reported that the γ process can be well described by a superposition of two Debye relaxation processes, which were attributed to the free water and the hydration water. The relaxation time of hydration water in that system was about four times larger than that of free water, which is corresponded with our results shown in Fig. 4(c). Note that dynamical behaviour of water geometrically confined in porous materials such as silica hydrogels and water in soft confinement systems including biomaterials in the deeply supercooled regime (*e.g.*, 150–230 K) has been investigated by the dielectric spectroscopy,^40–42^ in which two relaxation processes are often observed. A connection has been suggested between the two regimes and bimodal relaxation process of aqueous systems observed at room temperature.^42^

Molecular dynamics (MD) simulations previously performed in our lab^26^ have shown that rotational molecular dynamics of water strongly retarded within 3 Å from the protein surface, and the thickness of this hydration layer is independent of the concentration of protein. In addition, calculated average relaxation time of water in hydration layer (hydration water) has also only small dependency on the concentration of protein,^26^ with retaining the retardation factor for the hydration water about 5. The increase in the ratio for the intracellular slower water Δ*ε*_2_/(Δ*ε*_1_ + Δ*ε*_2_) shown in Fig. 3(b) reflects the increase in the ratio of the intracellular hydration water in more “crowded” environment of dehydrated cells, in which larger ratio of water interacts to biomolecules. On the other hand, the retardation factor for the relaxation time *τ*_2_/*τ*_1_ shown in Fig. 4(c) does not show a prominent change, which is corresponded with the concentration dependence of the relaxation time of hydration water and bulk-like water revealed by the MD simulations.^26^

## 4 Conclusions

We measured the molecular dynamics of water in dehydrated mammalian cells (Jurkat human T cells) by dielectric spectroscopy with a frequency up to 43.5 GHz. The dielectric spectra of the cells themselves were extracted by the measured complex permittivity of the cell suspension and the dispersion medium, based on the Bruggeman–Hanai equation. Two types of intracellular water—bulk-like water with a relaxation time *τ*_1_ of ∼10 ps and hydration water with a *τ*_2_ of ∼50 ps—were observed by analysis of the γ process around 10^10^ Hz. Our measurements revealed that the hydration water with slow water dynamics makes up a larger part of the water in dehydrated cells, while there is no significant change in the retardation factor between fast and slow relaxation times *τ*_2_/*τ*_1_. We anticipate that our technique for evaluating the intracellular water dynamics will be a helpful tool for understanding the effect of cryoprotective agents in the cryopreservation processes, by further examinations on various CPAs under broader temperature range.

## Author contributions

Hiroaki Matsuura: conceptualization; data curation; formal analysis; funding acquisition; investigation; methodology; resources; software; validation; visualization; writing – original draft; writing – review & editing. Kiyoshi Takano: resources. Ryo Shirakashi: conceptualization; funding acquisition; methodology; project administration; supervision; writing – review & editing.

## Conflicts of interest

There are no conflicts to declare.

## Supplementary Material

RA-013-D3RA02892E-s001

## References

[cit1] Ball P. (2008). Chem. Rev..

[cit2] Ball P. (2017). Proc. Natl. Acad. Sci. U. S. A..

[cit3] Toner M., Cravalho E. G. (1990). J. Appl. Phys..

[cit4] Zhmakin A. I. (2008). Phys.-Usp..

[cit5] Mazur P. (1963). J. Gen. Physiol..

[cit6] Raju R., Bryant S. J., Wilkinson B. L., Bryant G. (2021). Biochim. Biophys. Acta, Gen. Subj..

[cit7] Potma E. O., de Boeij W. P., Wiersma D. A. (2001). Biophys. J..

[cit8] Zaccai G. (2020). Biochim. Biophys. Acta, Gen. Subj..

[cit9] Tehei M., Franzetti B., Wood K., Gabel F., Fabiani E., Jasnin M., Zamponi M., Oesterhelt D., Zaccai G., Ginzburg M., Ginzburg B. Z. (2007). Proc. Natl. Acad. Sci. U. S. A..

[cit10] Stadler A. M., Embs J. P., Digel I., Artmann G. M., Unruh T., Büldt G., Zaccai G. (2008). J. Am. Chem. Soc..

[cit11] Piazza I., Cupane A., Barbier E. L., Rome C., Collomb N., Ollivier J., Gonzalez M. A., Natali F. (2018). Front. Phys..

[cit12] Jasnin M., Stadler A., Tehei M., Zaccai G. (2010). Phys. Chem. Chem. Phys..

[cit13] Persson E., Halle B. (2008). Proc. Natl. Acad. Sci. U. S. A..

[cit14] Sunde E. P., Setlow P., Hederstedt L., Halle B. (2009). Proc. Natl. Acad. Sci. U. S. A..

[cit15] Kaieda S., Setlow B., Setlow P., Halle B. (2013). Biophys. J..

[cit16] Tros M., Zheng L., Hunger J., Bonn M., Bonn D., Smits G. J., Woutersen S. (2017). Nat. Commun..

[cit17] Raicu V. and Feldman Y., Dielectric relaxation in biological systems: Physical principles, methods, and applications, Oxford University Press, New York, 2015

[cit18] Kaatze U., Feldman Y. (2006). Meas. Sci. Technol..

[cit19] Levy E., Barshtein G., Livshits L., Ishai P. B., Feldman Y. (2016). J. Phys. Chem. B.

[cit20] Levy E., David M., Barshtein G., Yedgar S., Livshits L., Ishai P. B., Feldman Y. (2017). J. Phys. Chem. B.

[cit21] Galindo C., Latypova L., Barshtein G., Livshits L., Arbell D., Einav S., Feldman Y. (2022). Eur. Biophys. J..

[cit22] Kraszewski A., Kulinski S., Matuszewski M. (1976). J. Appl. Phys..

[cit23] Ermolina I., Polevaya Y., Feldman Y., Ginzburg B.-Z., Schlesinger M. (2001). IEEE Trans. Dielectr. Electr. Insul..

[cit24] Hanai T. (1960). Kolloid-Z..

[cit25] Ebrahimi N., Sadeghi R. (2016). Fluid Phase Equilib..

[cit26] Hu K., Matsuura H., Shirakashi R. (2022). J. Phys. Chem. B.

[cit27] Matsuura H., Shirakashi R. (2022). Jpn. J. Appl. Phys..

[cit28] Wei L., Shirakashi R. (2020). J. Phys. Chem. B.

[cit29] Bruggeman D. A. G. (1935). Ann. Phys..

[cit30] Asami K. (2002). Prog. Polym. Sci..

[cit31] An Z., Kawashima D., Obara H., Takei M. (2021). IEEE Sens. J..

[cit32] Shinotsuka H., Yoshikawa H., Murakami R., Nakamura K., Tanaka H., Yoshihara K. (2020). J. Electron Spectrosc. Relat. Phenom..

[cit33] Asami K. (2007). J. Phys. D: Appl. Phys..

[cit34] Ron A., Fishelson N., Croitoriu N., Benayahu D., Shacham-Diamand Y. (2009). Biophys. Chem..

[cit35] Weingärtner H., Knocks A. (2001). J. Chem. Phys..

[cit36] Perticaroli S., Nakanishi M., Pashkovski E., Sokolov A. P. (2013). J. Phys. Chem. B.

[cit37] Shiraga K., Adachi A., Nakamura M., Tajima T., Ajito K., Ogawa Y. (2017). J. Chem. Phys..

[cit38] Braun D., Schmollngruber M., Steinhauser O. (2017). Phys. Chem. Chem. Phys..

[cit39] Robles-Hernández B., González E., Pomposo J. A., Colmenero J., Alegría A. (2020). Soft Matter.

[cit40] Cerveny S., Mallamace F., Swenson J., Vogel M., Xu L. (2016). Chem. Rev..

[cit41] Breynaert E., Houlleberghs M., Radhakrishnan S., Grübel G., Taulelle F., Martens J. A. (2020). Chem. Soc. Rev..

[cit42] Cerveny S., Alegría Á., Colmenero J. (2008). Phys. Rev. E: Stat., Nonlinear, Soft Matter Phys..

