# Supplementary material for: Slow water dynamics in dehydrated human Jurkat T cells evaluated by dielectric spectroscopy with the Bruggeman–Hanai equation
Source: RSC Adv. 2023 Jul 11;13(30):20934–40. doi: 10.1039/d3ra02892e (PMC10334875; doi:10.1039/d3ra02892e)
Supplement: RA-013-D3RA02892E-s001 [file RA-013-D3RA02892E-s001.pdf]

Electronic Supplementary Material (ESI) for RSC Advances.
This journal is © The Royal Society of Chemistry 2023

Supplementary information for

Water slow dynamics in dehydrated human Jurkat T cells evaluated by dielectric spectroscopy with Bruggeman–Hanai equation

Hiroaki Matsuura[1,2]*, Kiyoshi Takano[1], and Ryo Shirakashi[1]

[1]*Institute of Industrial Science, The University of Tokyo, Meguro, Tokyo 153-8505, Japan*

[2]*Research Fellow of the Japan Society for the Promotion of Science, Chiyoda, Tokyo 102-0083, Japan*

E-mail: matsuura@iis.u-tokyo.ac.jp

Measurement of volume fraction of cells

Jurkat cells suspended in aqueous sucrose solutions were observed by a confocal laser fluorescence microscope (FV-300, Olympus, Japan). For the fluorescence observation of cells, a small volume of the aqueous solution of Rhodamine B (Fujifilm Wako Pure Chemical, Japan) was added to the cell suspension. The cell suspension was put into the well with the depth of 0.35 mm and covered by the cover glass. The well for the microscopic observation was prepared by adhering a flat ring of metal on a slide glass. Although the dimension of the well is different from the test fixture for the DS measurement, we assume that the volume fraction of the cells in the DS sensing volume can be estimated by the confocal microscopic observation, for the concentrated samples used in the present study. The XYZ image acquisitions were repeated at several points. Fluorescence images of cell suspensions were analyzed by the image processing program of ImageJ,[1] based on the auto thresholding algorithm by Li.[2]

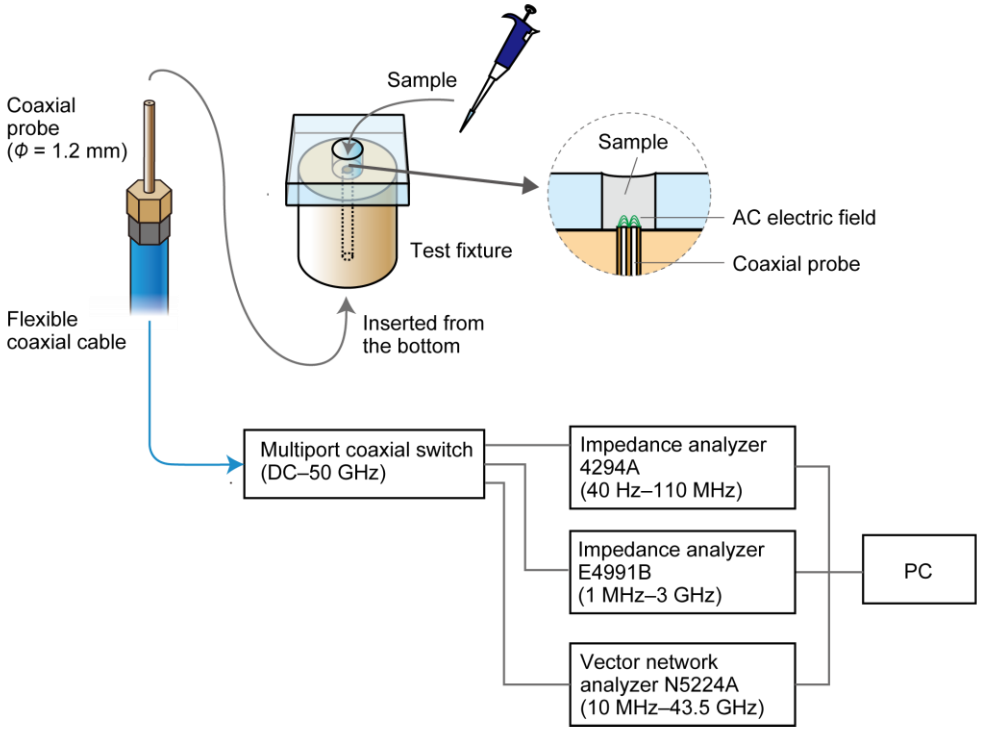

Fig.S1. The experimental setup for the dielectric spectroscopy. The multiport coaxial switch enables the measurement of the same sample by three instruments without reconnecting cables. In the experiments, the dielectric spectra of 500 Hz–110 MHz, 1 MHz–3 GHz, and 200 MHz–43.5 GHz were obtained by the impedance analyzer 4294A, the impedance analyzer E4991B, and vector network analyzer N5224A, respectively. Note that a vector network analyzer is not suitable for the measurement of the permittivity below several hundreds of MHz because the impedance of a sample is much larger than the specific impedance.[3] To cover that frequency range, the impedance analyzer E4991B based on another measurement principle (RF I-V method) was employed. The dielectric spectra by three instruments were connected at overlapped frequency range. A lab-made test fixture (dielectric cell) was designed for the dielectric spectroscopy of cell suspensions in this study. A cylindrical brass and an acrylic resin plate with a cylindrical hole are bonded together with epoxy adhesive to compose a fixture to fill the sample liquid (Fig.S1). A part of this test fixture is made of metal (brass) for a better temperature controlling. A coaxial probe electrode with characteristic impedance of 50 Ohm is inserted into the bottom hole of this test fixture. The sample is put into the top hole of the test fixture, with the volume of about 20 μl. The electric field for measurement is applied between the center and the surrounding conductor of the coaxial probe to form a part of the circuit in measuring system. We calibrated this test fixture circuit using three standard materials with known dielectric properties (air, ethylene glycol, water).

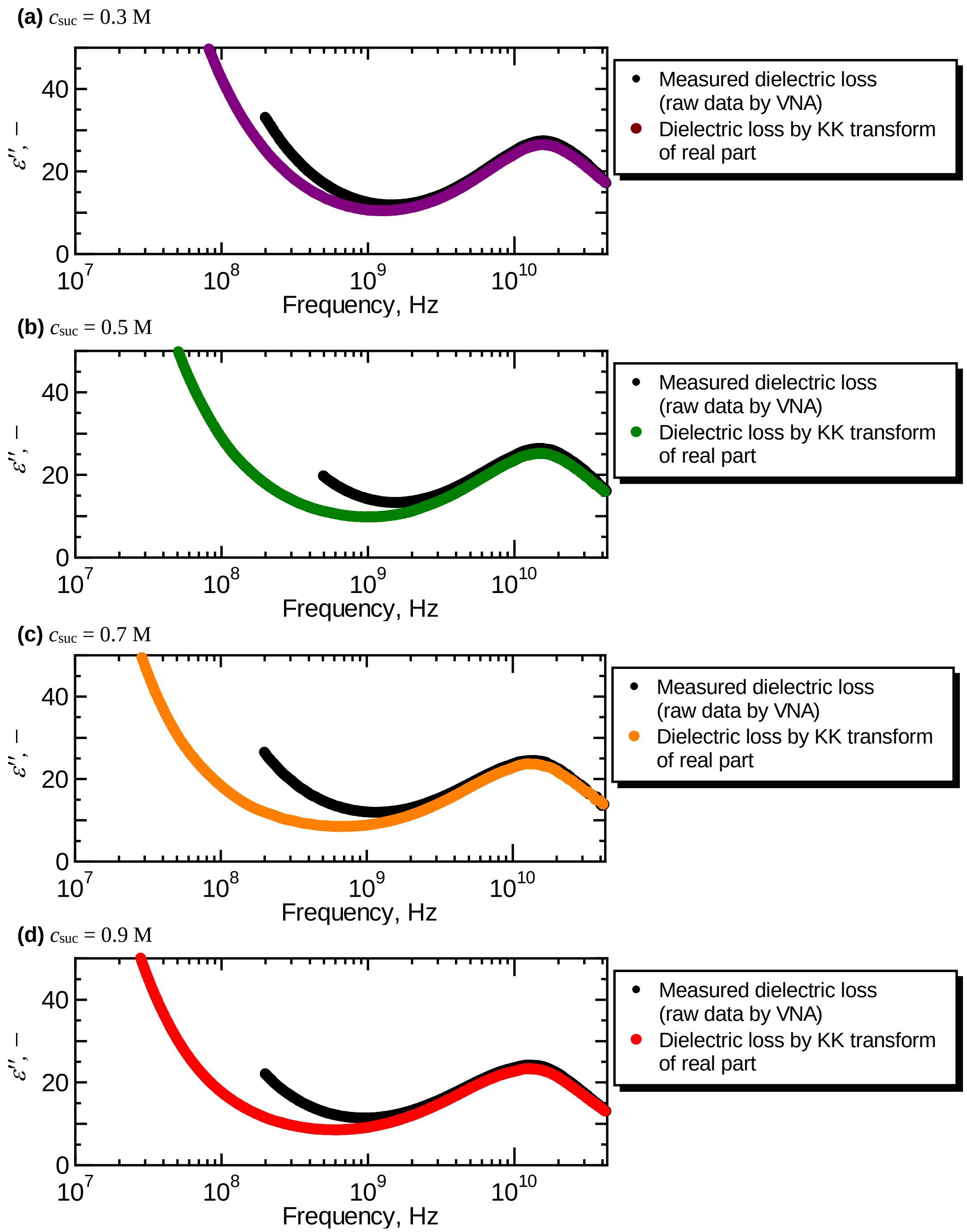

Fig. S2. Comparison of the dielectric loss spectrum obtained by the Kramers–Kronig transform of the real part and that directly obtained by the vector network analyzer (VNA), which contains the contribution of the Ohmic loss.

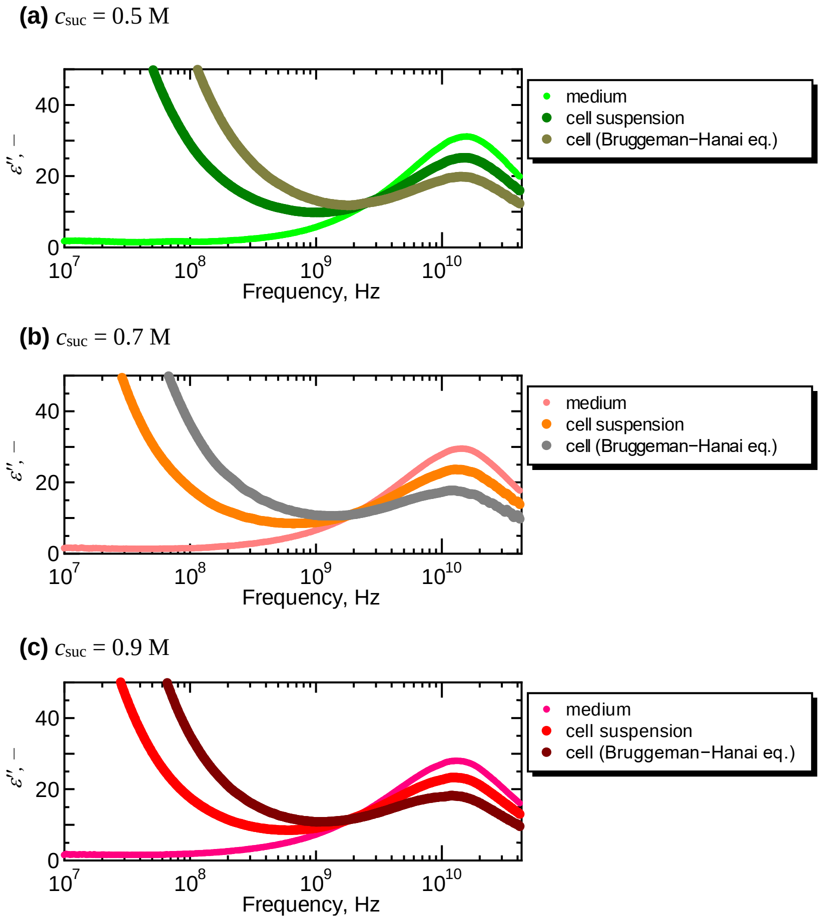

Fig. S3. The dielectric loss spectra for dispersion medium, cell suspension, and cells (by Bruggeman-Hanai equation) for the sucrose aqueous solutions of (a) c_{suc} = 0.5 M, (b) c_{suc} = 0.7 M, (c). c_{suc} = 0.9 M.

(a) c_{suc} = 0.3 M

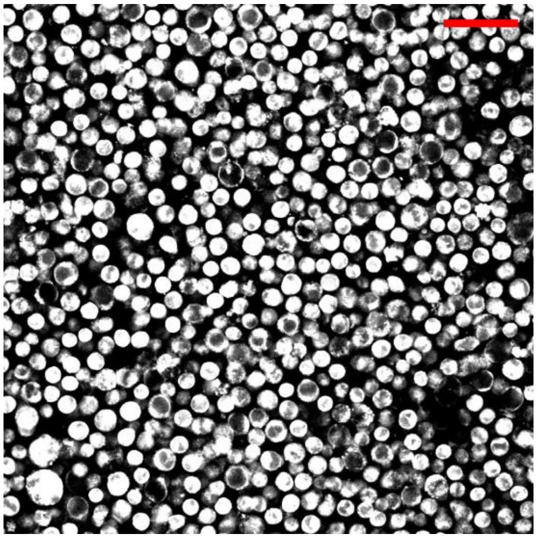

(b) c_{suc} = 0.5 M

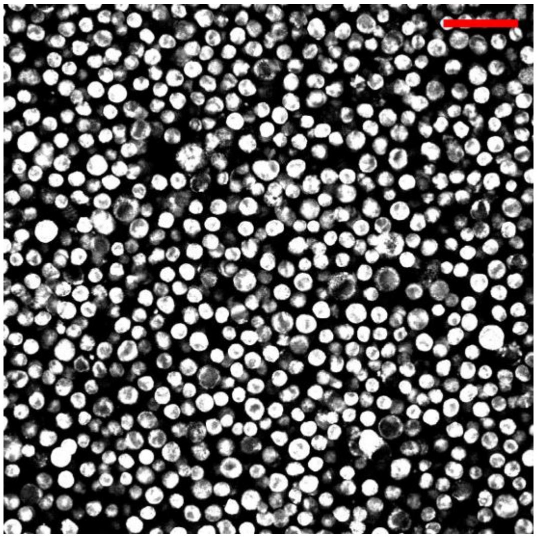

(c) c_{suc} = 0.7 M

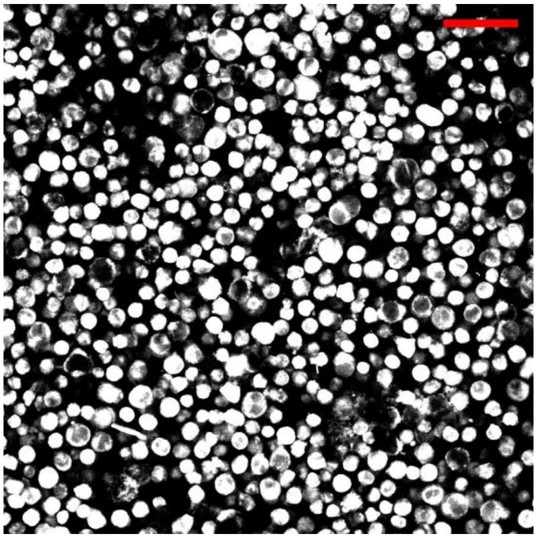

(d) c_{suc} = 0.9 M

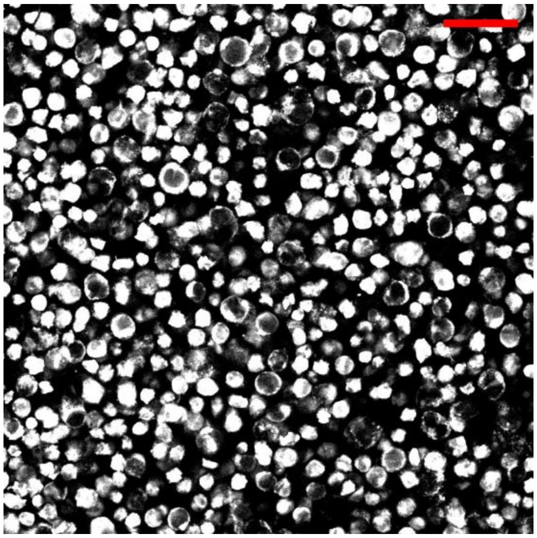

Fig. S4 Jurkat cells suspended in aqueous sucrose solutions observed by a confocal laser fluorescence microscope. Fluorescence images were analyzed by the image processing program of ImageJ,[1] based on the auto thresholding algorithm by Li.[2] The scale bar indicates 50 μm.

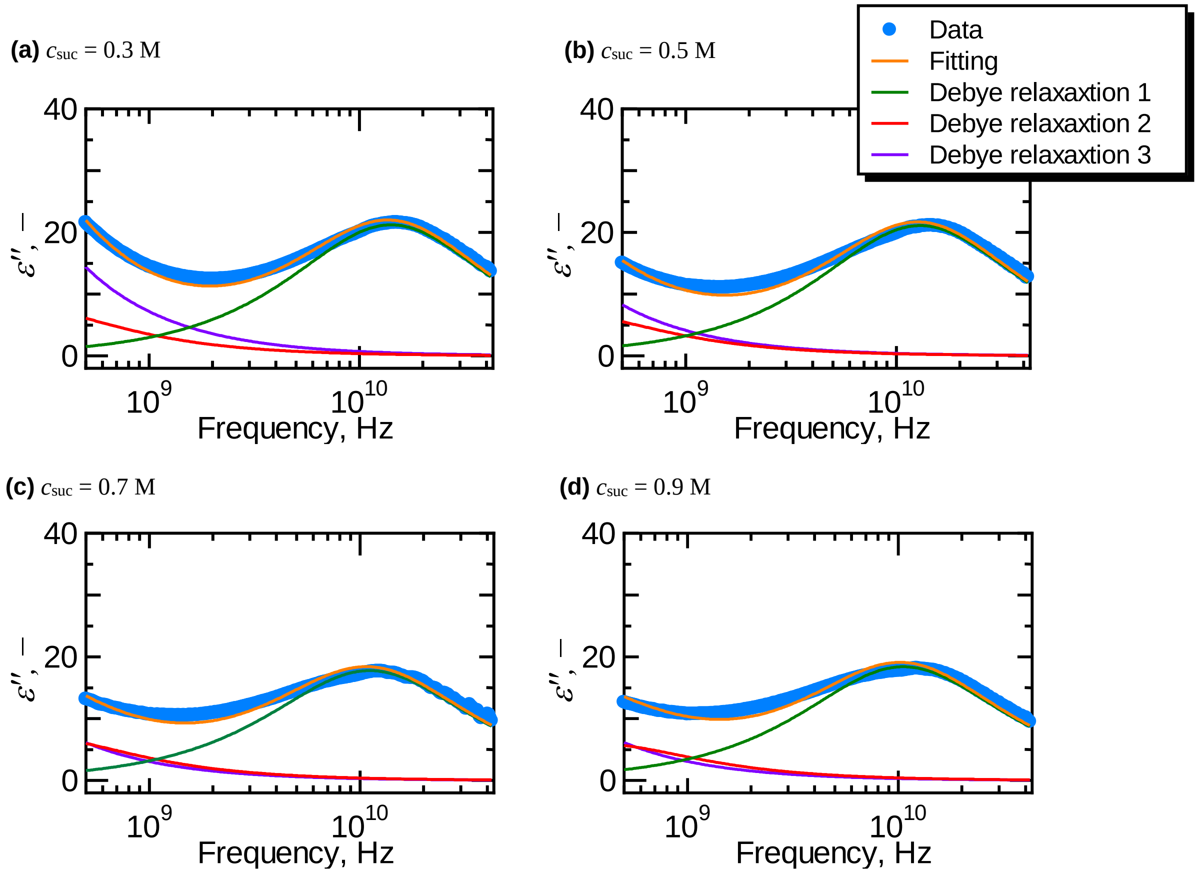

Fig. S5. Fitting to intracellular γ process by a single Debye relaxation.

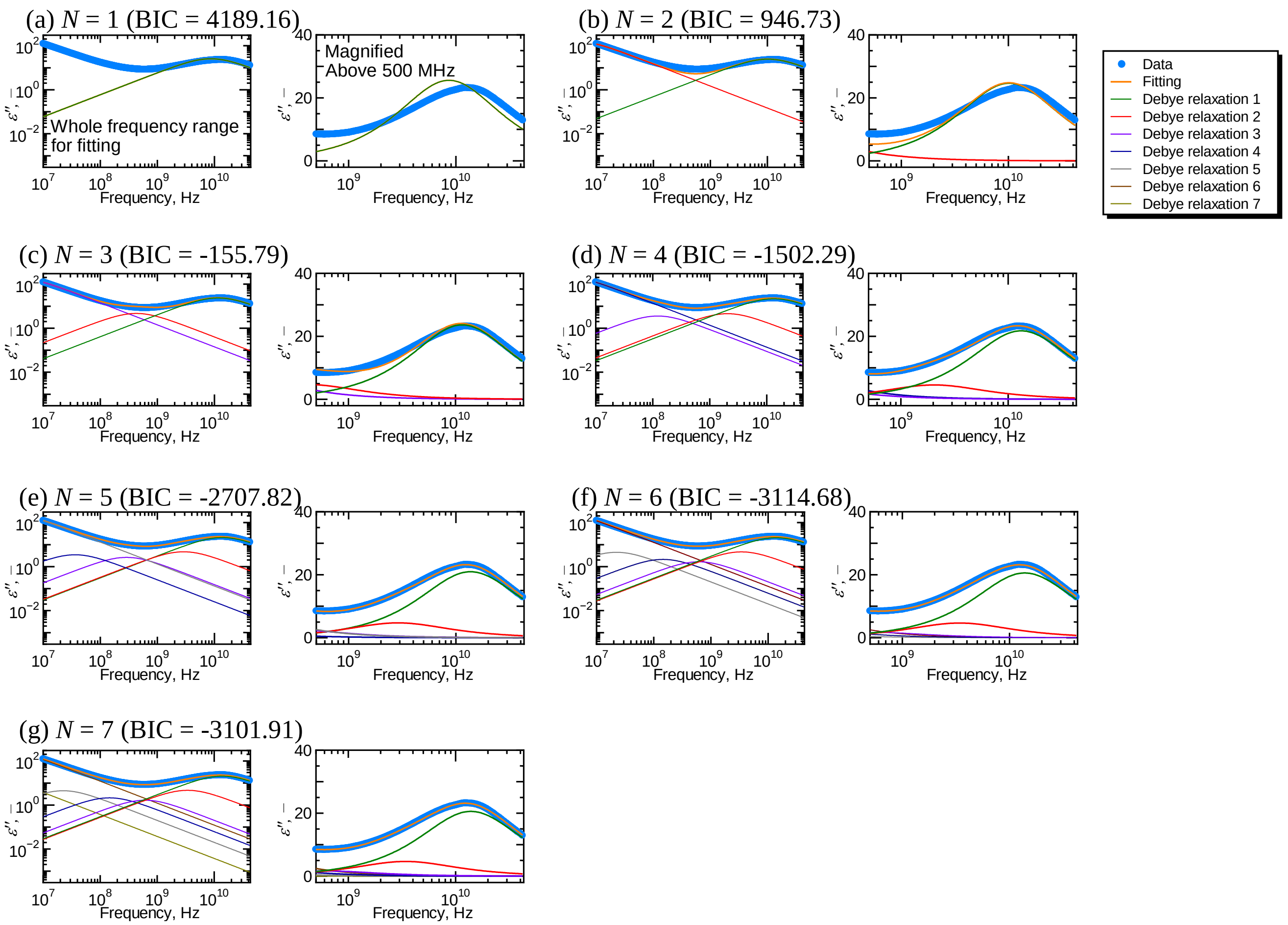

Fig. S6. Bayesian information criterion (BIC) for $N = 1, 2, \ldots, 7$. (spectrum of cell suspension with c_{suc} = 0.9 M) in the decomposition of spectrum into sum of Debye relaxations.

Table S1. Fitting parameters based on BIC in the decomposition of spectra into sum of Debye relaxations.

	c_{suc}	Number of relaxation terms N	Fitting parameters	
			Relaxation strength $\Delta\varepsilon_n$	Relaxation time τ_n
Dispersion medium	0.3 M	2	$\Delta\varepsilon_1$, $\Delta\varepsilon_2$	τ_1, τ_2
	0.5 M	2	$\Delta\varepsilon_1$, $\Delta\varepsilon_2$	τ_1, τ_2
	0.7 M	3	$\Delta\varepsilon_1$, $\Delta\varepsilon_2$, $\Delta\varepsilon_3$	τ_1, τ_2, τ_3
	0.9 M	3	$\Delta\varepsilon_1$, $\Delta\varepsilon_2$, $\Delta\varepsilon_3$	τ_1, τ_2, τ_3
Cell suspensions	0.3 M	6	$\Delta\varepsilon_1$, $\Delta\varepsilon_2$, $\Delta\varepsilon_3$, $\Delta\varepsilon_4$, $\Delta\varepsilon_5$	τ_1, τ_2, τ_3, τ_4, τ_5
	0.5 M	6	$\Delta\varepsilon_1$, $\Delta\varepsilon_2$, $\Delta\varepsilon_3$, $\Delta\varepsilon_4$, $\Delta\varepsilon_5$, $\Delta\varepsilon_6$	τ_1, τ_2, τ_3, τ_4, τ_5, τ_6
	0.7 M	6	$\Delta\varepsilon_1$, $\Delta\varepsilon_2$, $\Delta\varepsilon_3$, $\Delta\varepsilon_4$, $\Delta\varepsilon_5$, $\Delta\varepsilon_6$	τ_1, τ_2, τ_3, τ_4, τ_5, τ_6
	0.9 M	6	$\Delta\varepsilon_1$, $\Delta\varepsilon_2$, $\Delta\varepsilon_3$, $\Delta\varepsilon_4$, $\Delta\varepsilon_5$, $\Delta\varepsilon_6$	τ_1, τ_2, τ_3, τ_4, τ_5, τ_6
Cell	0.3 M	5	$\Delta\varepsilon_1$, $\Delta\varepsilon_2$, $\Delta\varepsilon_3$, $\Delta\varepsilon_4$, $\Delta\varepsilon_5$	τ_1, τ_2, τ_3, τ_4, τ_5
	0.5 M	5	$\Delta\varepsilon_1$, $\Delta\varepsilon_2$, $\Delta\varepsilon_3$, $\Delta\varepsilon_4$, $\Delta\varepsilon_5$	τ_1, τ_2, τ_3, τ_4, τ_5
	0.7 M	5	$\Delta\varepsilon_1$, $\Delta\varepsilon_2$, $\Delta\varepsilon_3$, $\Delta\varepsilon_4$, $\Delta\varepsilon_5$	τ_1, τ_2, τ_3, τ_4, τ_5
	0.9 M	5	$\Delta\varepsilon_1$, $\Delta\varepsilon_2$, $\Delta\varepsilon_3$, $\Delta\varepsilon_4$, $\Delta\varepsilon_5$	τ_1, τ_2, τ_3, τ_4, τ_5

Table S2. Repeatability of the fitting results for one spectrum of cell suspension (c_{suc} = 0.9 M) with different initial values of τ_n.*

	Run 1		Run 2		Run 3	
	Init. values	Fitting results	Init. values	Fitting results	Init. values	Fitting results
$\Delta\varepsilon_1$	50	41.13	50	41.13	50	41.13
$\Delta\varepsilon_2$	10	9.37	10	9.37	10	9.37
$\Delta\varepsilon_3$	1	3.31	1	3.31	1	3.31
$\Delta\varepsilon_4$	1	4.26	1	4.26	1	4.26
$\Delta\varepsilon_5$	1	8.82	1	8.82	1	8.82
$\Delta\varepsilon_6$	1	571.14	1	571.14	1	571.14
τ_1	8.00×10^{-12}	1.1488×10^{-11}	8.00×10^{-12}	1.1488×10^{-11}	8.00×10^{-12}	1.1488×10^{-11}
τ_2	2.00×10^{-11}	4.6606×10^{-11}	2.00×10^{-11}	4.6606×10^{-11}	2.00×10^{-11}	4.6606×10^{-11}
τ_3	3.47×10^{-10}	2.5852×10^{-10}	5.03×10^{-7}	2.5852×10^{-10}	3.02×10^{-7}	2.5852×10^{-10}
τ_4	1.15×10^{-6}	1.0922×10^{-9}	6.26×10^{-9}	1.0922×10^{-9}	3.87×10^{-8}	1.0922×10^{-9}
τ_5	1.24×10^{-9}	7.0170×10^{-9}	3.16×10^{-8}	7.0170×10^{-9}	3.54×10^{-11}	7.0170×10^{-9}
τ_6	4.37×10^{-7}	7.1954×10^{-8}	4.65×10^{-8}	7.1954×10^{-8}	1.13×10^{-7}	7.1954×10^{-8}

	Run 4		Run 5	
	Init. values	Fitting results	Init. values	Fitting results
$\Delta\varepsilon_1$	50	41.13	50	41.13
$\Delta\varepsilon_2$	10	9.37	10	9.37
$\Delta\varepsilon_3$	1	3.31	1	3.31
$\Delta\varepsilon_4$	1	4.26	1	4.26
$\Delta\varepsilon_5$	1	8.82	1	8.82
$\Delta\varepsilon_6$	1	571.14	1	571.14
τ_1	8.00×10^{-12}	1.1488×10^{-11}	8.00×10^{-12}	1.1488×10^{-11}
τ_2	2.00×10^{-11}	4.6606×10^{-11}	2.00×10^{-11}	4.6606×10^{-11}
τ_3	1.66×10^{-7}	2.5852×10^{-10}	2.85×10^{-9}	2.5852×10^{-10}
τ_4	2.96×10^{-10}	1.0922×10^{-9}	3.27×10^{-10}	1.0922×10^{-9}
τ_5	1.27×10^{-10}	7.0170×10^{-9}	6.40×10^{-8}	7.0170×10^{-9}
τ_6	1.25×10^{-11}	7.1954×10^{-8}	1.65×10^{-9}	7.1954×10^{-8}

*Initial values of the relaxation strength for the fitting were $\Delta\varepsilon_{1\_init}$ = 50, $\Delta\varepsilon_{2\_init}$ = 10, and $\Delta\varepsilon_{n\_init}$ = 1 (n > 2) for all samples in this study. For the relaxation time, initial values for first and second process were $\tau_{1\_init} = 8.00 \times 10^{-12}$ s and $\tau_{2\_init} = 2.00 \times 10^{-11}$ s. τ_{n_init} (n > 2) were determined randomly between 8×10^{-12} s and 1×10^{-4} s using "numpy.random.rand" method of Python NumPy. These initial values were determined for the efficiency of the fitting analysis, after we confirmed that the fitting results with two Debye relaxations for γ process are reasonable based on BIC. Note that γ process obtained in this study is decomposed into two Debye relaxations with different initial values of $\Delta\varepsilon_{1\_init}$, $\Delta\varepsilon_{2\_init}$, τ_{1_init}, and τ_{2_init}.

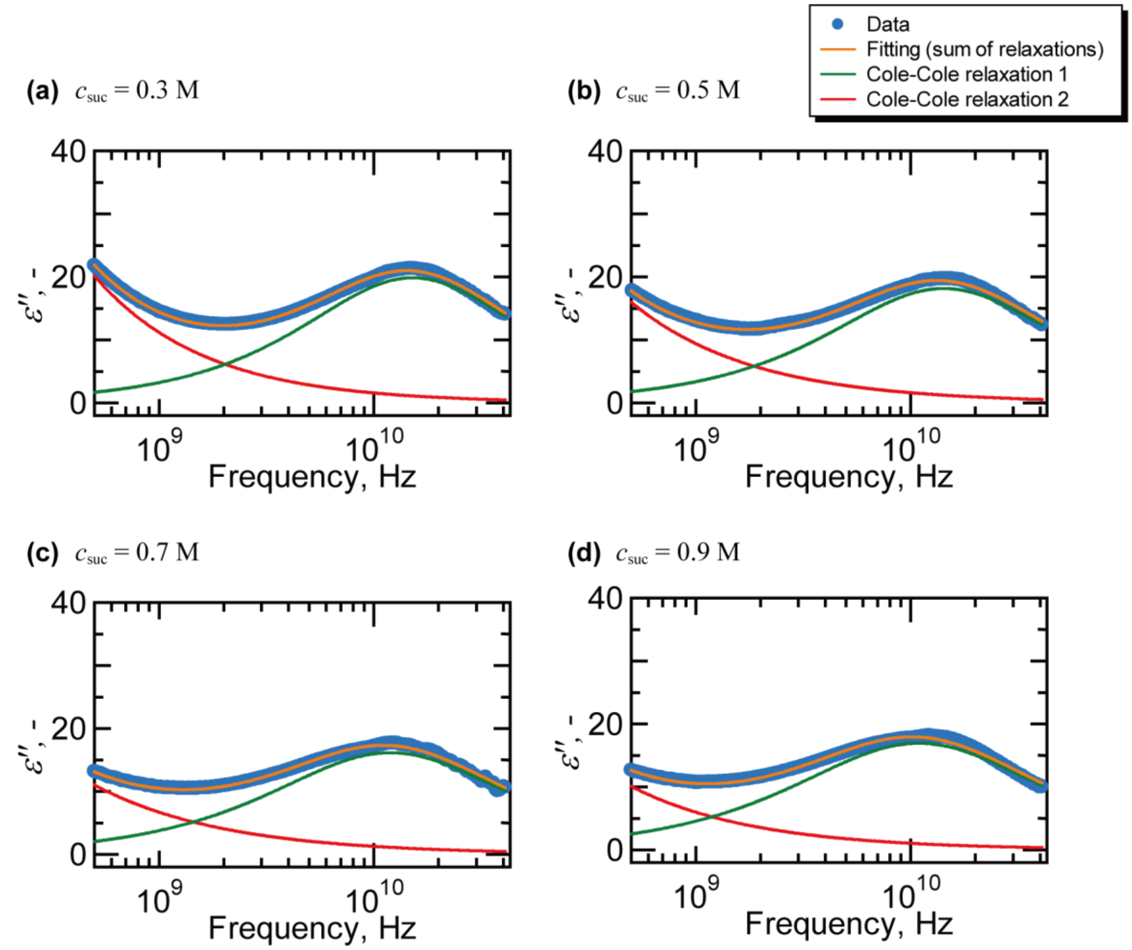

Fig. S7. Decomposition of the dielectric loss spectra based on the Cole-Cole relaxation functions for cells (by Bruggeman-Hanai equation). The fitting analysis was performed on the dielectric loss spectra with the frequency range of 100 MHz to 43.5 GHz. The fitting results above 500 MHz are shown for the visual convenience of γ process.

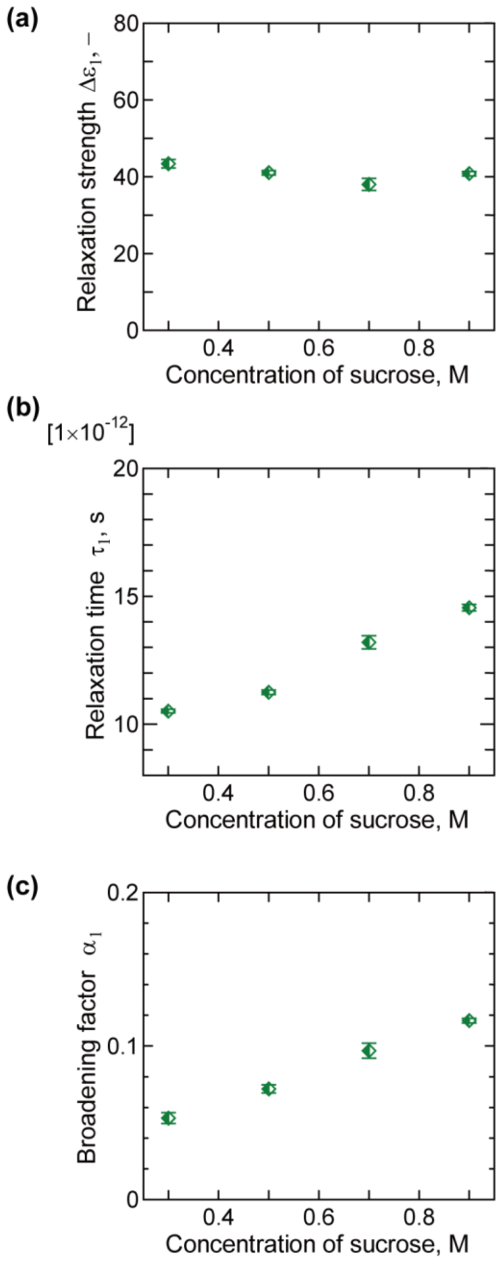

Fig. S8. Results of the fitting to the spectra of cells (by Bruggeman-Hanai equation), based on the Cole-Cole relaxation functions (Fig. S7). (a) Relaxation strength of 1st Cole-Cole relaxation $\Delta\varepsilon_1$. (b) Relaxation time τ_1. (c) Broadening factor α_1. Contribution of the uncertainty of the measurement of volume fraction is combined.

References

1) M. D. Abràmoff, P. J. Magelhaes, S. J. Ram, Image processing with ImageJ, Biophotonics Int., 11, 36 (2004).

2) C. H. Li, C. K. Lee, Minimum cross entropy thresholding, Pattern Recognit., 26, 617 (1993).

3) Keysight Technologies, Impedance Measurement Handbook, Application note 5950-3000, (2020).
